# Multimodal artificial intelligence in medicine: a task-oriented framework for clinical translation

**DOI:** 10.3389/fmed.2025.1736272

**Published:** 2026-01-14

**Authors:** Ruiying Zhang, Yan Chen, Wen Yue, Yi Zhang, Xin Li, Shuo Feng, Feng Yuan, Mingran Luo

**Affiliations:** 1 Department of Orthopedics, The Affiliated Hospital of Xuzhou Medical University, Xuzhou, China; 2 MOE Key Laboratory for Biomedical Photonics, Wuhan National Laboratory for Optoelectronics, Huazhong University of Science and Technology, Wuhan, China; 3 School of Medical Imaging, Henan Medical University, Xinxiang, China; 4 Department of Orthopedics, The Affiliated Lianyungang Hospital of Xuzhou Medical University (The First People’s Hospital of Lianyungang), Lianyungang, China

**Keywords:** AI diagnosis, clinical applications, data fusion, multimodal AI, personalized therapy, precision medicine

## Abstract

Multimodal artificial intelligence (AI) technologies are transforming medical practices by integrating diverse data sources to enable more accurate diagnosis, disease prediction, and treatment planning. In this review, we explore state-of-the-art multimodal AI systems, focusing on their applications in clinical settings, including radiology, pathology, and clinical imaging, as well as non-image data, such as electronic health records (EHRs) and multi-omics data. We highlight how combining multiple modalities improves diagnostic accuracy and prognostic prediction compared to unimodal models. The study emphasizes the importance of robust data fusion strategies and model interpretability for real-world clinical deployment. By addressing key challenges, such as data heterogeneity and uncertainty quantification, this research offers a new paradigm for intelligent healthcare. The findings suggest that the continued advancement of multimodal AI will significantly enhance clinical decision-making, paving the way for personalized medicine and improved patient outcomes.

## Introduction

As the data-driven paradigm continues to evolve, the medical and healthcare fields are undergoing an unprecedented transformation. An increasing number of diagnostic, therapeutic, and research processes are systematically shifting toward data-centric models, substantially enhancing the precision of clinical decision-making and the evidence-based foundation of health management (1). This shift has been driven by the multidimensional expansion of medical data sources, including the widespread adoption of electronic health record (EHR) systems (2), breakthroughs in multi-omics technologies (3), and the large-scale implementation of smart wearable devices (4). These diverse data modalities offer novel perspectives for elucidating disease phenotypes. Multi-source heterogeneous data exhibit significant dimensional variability and rich expressiveness. These characteristics enable the extraction of complementary biosignatures across multiple biological scales, including structural morphology, functional dynamics, and molecular regulatory networks, providing a more holistic characterization of health at both individual and population levels (5). Such integration enhances disease screening sensitivity, refines risk stratification, and improves treatment response prediction, ultimately driving the clinical applicability of precision medicine. As biomedical research advances, there is an urgent need for computationally efficient and statistically robust frameworks capable of unlocking the full potential of multimodal data and translating it into actionable clinical insights.

Traditional clinical workflows predominantly employ a linear, manual approach to multimodal data integration. Clinicians are required to reconstruct fragmented information into a coherent clinical profile by retrieving and synthesizing disparate data across multiple systems—such as EHRs, laboratory information systems (LIS), and picture archiving and communication systems (PACS). However, as the volume of medical data increases exponentially and its heterogeneity intensifies, this fragmented, labor-intensive integration process faces mounting challenges. Significant challenges now include diminished clinical efficiency (6), escalating cognitive load for healthcare providers (7), and elevated risks of overlooking critical diagnostic information (8, 9), collectively impairing both the timeliness and accuracy of clinical decision-making. To address these limitations, artificial intelligence (AI)-driven strategies have emerged as a promising direction in clinical informatics research (10–12).

To date, most medical AI applications remain focused on models built using unimodal data sources such as medical imaging, structured clinical records, or genomic profiles (13–15), which often limits the effective integration of the rich, heterogeneous information embedded in clinical workflows. This unimodal focus may compromise the generalizability of AI models in real-world settings and reduce their ability to comprehensively reflect a patient’s health status. As a result, such models are more prone to information loss or representation gaps, which can contribute to diagnostic uncertainty or suboptimal decision-making in complex cases (16, 17). For instance, medical imaging primarily captures morphological lesion characteristics; electronic health records (EHRs) document longitudinal treatment histories and laboratory findings; while genomic data uncover the molecular underpinnings of disease at the individual level. Although each modality contains valuable and often high-dimensional information, analyzing them in isolation frequently neglects the potential complementarities and cross-modal relationships among these heterogeneous data types. Recent empirical studies have shown that integrating multiple modalities - under controlled modeling conditions - can significantly improve diagnostic accuracy and robustness compared to unimodal baselines (18, 19). These limitations also differ substantially across clinical tasks—for instance, a single imaging exam or physiological signal often cannot provide timely and stable early warning in monitoring settings, whereas image-only models struggle to capture molecular heterogeneity or comorbidities in treatment planning and prognosis. Such task-specific shortcomings motivate the need for a task-oriented framework that explicitly links multimodal design choices to the requirements of different clinical decisions.

Recent advances have demonstrated that multimodal AI models—which integrate data from diverse sources such as medical images, clinical narratives, and laboratory test results—can construct unified representations that significantly enhance predictive performance across a range of diagnostic and therapeutic tasks. These models offer a more comprehensive evidence base for clinical decision-making and have shown superior accuracy and robustness compared to their unimodal counterparts, thus holding substantial promise for both translational research and clinical implementation (20, 21). The key advantage of multimodal approaches lies in their ability to leverage complementary information and perform collaborative reasoning across modalities, thereby capturing disease heterogeneity at multiple biological and clinical levels—including structural, molecular, and phenotypic dimensions. This integrative capacity enables the development of prediction models that are not only more biologically informative but also more clinically actionable (22, 23).

The landscape of multimodal medical AI is undergoing a paradigm shift—from task-specific, small-scale models focused on individual disease domains to large-scale architectures designed to integrate diverse data sources within unified frameworks. This shift reflects the growing demand for clinical decision support systems (CDSS) that are real-time, interpretable, and capable of leveraging heterogeneous medical data. To address this need, we propose a novel task-oriented synthesis framework that organizes the field along three axes: (1) a systematic overview of multimodal AI methodologies; (2) an in-depth analysis of clinical decision-making scenarios, including diagnosis, treatment planning, and patient monitoring; and (3) an evaluation of interdisciplinary applications beyond traditional clinical tasks. This structure provides a comprehensive roadmap that directly links methodological advances to clinical practice.

Unlike previous reviews, which often emphasize either disease-specific applications (24, 25), or methodological details in isolation (26, 27), our synthesis bridges these perspectives by integrating technical rigor with clinical relevance. Supported by evidence summarised in our comparative analyses, multimodal AI systems generally show improved predictive accuracy, generalizability, and clinical utility relative to unimodal baselines. By incorporating imaging, physiological signals, histopathology, and clinical narratives, these systems enable holistic characterization of disease phenotypes and improved modeling of complex mechanisms. Furthermore, the integration of generative AI, particularly large language models (LLMs), alongside reinforcement learning and foundation models, is accelerating the development of intelligent, interpretable, and interactive system architectures.

However, a critical translational gap persists: despite rapid technological progress, the clinical adoption of multimodal AI remains limited. We posit that the primary barrier lies in a fundamental misalignment—many sophisticated models are optimized for isolated benchmark performance rather than for addressing the integrated and pragmatic demands of real clinical workflows. To bridge this gap, this review introduces a task-oriented framework. We argue that the design, fusion strategy, and evaluation of multimodal AI must be intrinsically guided by the concrete objectives of clinical practice, from diagnosis and treatment planning to population-level health surveillance. It is through this task-oriented lens that the advances discussed herein can establish a robust foundation for precision medicine, public health, and interdisciplinary biomedical research. Building upon this perspective, we highlight the emerging role of mechanism-aware integration as a bridge between methodological innovation and clinical translation, pointing toward a more interpretable and biologically grounded paradigm for multimodal medical AI.

## Technical landscape of multimodal AI in medicine

Over the past several years, research on multimodal AI in medicine has gradually grown, with an observable shift from single-modality models toward heterogeneous data integration. As diverse medical data (imaging, omics, EHRs) and deep learning techniques have become more accessible, a growing body of publications illustrates increasing interest in multimodal AI and a widely perceived potential for clinical application.

We conducted a focused literature search on original research articles reporting multimodal AI methods for clinical or public-health applications. PubMed, Web of Science, Embase, and Scopus were queried for the period January 2020–August 2025 using predefined combinations of multimodal-AI–related and medical/task-specific keywords connected by Boolean operators (AND/OR), and the results were complemented by manual screening of reference lists from key multimodal AI and survey papers. The methodological quality and clinical relevance of the included studies were qualitatively considered by two domain experts in our team, with disagreements resolved by discussion and consensus.

Building upon a synthesis of existing literature, this chapter focuses on the common data modalities, representation learning strategies, fusion techniques, and model architectures employed in multimodal medical AI research. The structure of this paper is organized as follows: Chapter 3 will present a detailed survey of recent applications of multimodal AI across a spectrum of medical tasks, while Chapter 4 will offer a critical discussion of research outcomes and a comprehensive analysis of the key technical challenges and future directions for clinical translation.

## Data modalities and medical task landscape

**Figure 1:**
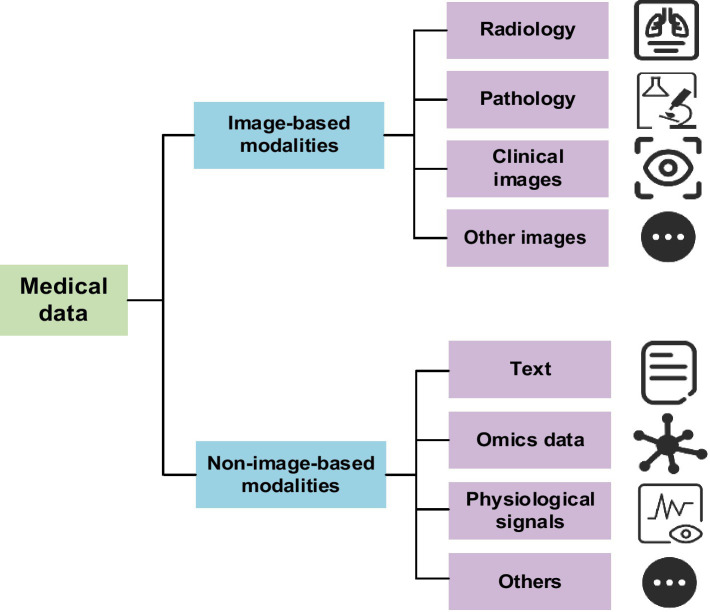
The hierarchical classification of medical data modalities, which serves as the foundation for multimodal AI applications in healthcare.

In the reviewed body of research on multimodal medical AI, the types of data employed can be broadly categorized into two main modalities: imaging and non-imaging data (Figure 1). Imaging modalities are typically classified according to their clinical specialty, encompassing radiological imaging (e.g., CT, MRI, ultrasound, X-ray, and nuclear medicine imaging), pathological imaging (e.g., histopathological slides), and general clinical imaging (e.g., optical coherence tomography [OCT], fundus photography, and dermatological images). For certain image types with limited sample sizes in the literature, this review consolidates them under the category of “other imaging modalities” for analytical consistency.

In contrast to image-based modalities, non-image data encompass a wide spectrum of structured and unstructured formats, including but not limited to laboratory test results, EHRs, and multi-omics data such as genomics, transcriptomics, and proteomics. Additionally, physiological signals (e.g., electroencephalography [EEG], electrocardiography [ECG]) and continuous data streams from wearable devices (e.g., heart rate, blood oxygen saturation, physical activity trajectories) are increasingly integrated into clinical research. These diverse modalities are often flexibly combined in multimodal frameworks to provide a more comprehensive and multidimensional representation of a patient’s medical state, thereby enhancing the robustness and predictive capability of computational models in healthcare applications.

According to the reviewed studies, radiomics and textual data are the most commonly utilized data modalities, followed by omics and pathology data. There is a clear preference for certain modality combinations, with the integration of radiomics and textual data being the most prevalent. This reflects the complementary role of imaging and clinical text in supporting diagnosis and disease assessment. Additionally, combinations such as pathology with omics, and clinical images with textual data, have also been adopted to some extent, highlighting the potential of multimodal integration for fusing information at both the tissue and molecular levels. Although the use of three or more modalities remains relatively limited, emerging attempts to integrate radiology, pathology, omics, and textual data suggest a growing trend toward the development of cross-scale integrative models.

The literature review indicates that multimodal AI has been widely applied across various major disease types, with particular emphasis on oncological diseases (e.g., lung cancer, breast cancer, colorectal cancer), neurological disorders (e.g., Alzheimer’s disease, Parkinson’s disease), and cardiovascular and metabolic conditions (e.g., coronary artery disease, diabetes). Among these, cancer-related diseases have become a major focus of multimodal AI research due to their high heterogeneity and the abundance of high-dimensional data. Studies often integrate radiological imaging, pathological slides, omics data, and clinical records to enhance the accuracy and interpretability of AI models in tasks such as disease subtyping, survival prediction, and treatment response assessment (28). For chronic and complex conditions such as neurodegenerative diseases, longitudinal follow-up data and physiological signals are more frequently utilized, where the integration of structural imaging and time-series information enables the development of dynamic prediction models (29, 30).

In terms of medical applications, multimodal AI primarily supports several core tasks, including disease diagnosis and clinical decision support, patient monitoring and telemedicine, patient self-management and health maintenance, as well as public and population health surveillance. Among these, disease diagnosis remains the predominant focus of current research, particularly in the contexts of oncology and chronic diseases. Multimodal approaches enable the integration of heterogeneous information across different biological and clinical levels—from macro-scale imaging to molecular-level mechanisms - thereby improving both the clinical relevance and robustness of predictive models. While many existing studies still employ single-task frameworks, there is a growing trend toward multi-task learning. In complex scenarios such as tumor subtyping and treatment pathway optimization (31), multimodal AI demonstrates unique advantages in integrating diverse sources of biomedical information.

## Feature encoding and fusion strategies

In multimodal medical AI systems, feature encoding serves as the initial step in the processing pipeline, transforming modality-specific data into representations suitable for integration and interpretation. For imaging modalities such as radiology and pathology, convolutional neural networks (CNNs) and Vision Transformers (ViTs) are widely used to extract spatial features. Textual data employ pretrained language models like BERT (32) and ClinicalBERT (33) to capture contextual semantics, while structured clinical variables and multi-omics data are often encoded with multilayer perceptrons (MLPs) or graph neural networks (GNNs). Recently, self-supervised learning methods [e.g., SimCLR (34), DINO (35), and Masked Autoencoders (36)] have been adopted for pretraining on unlabeled datasets, improving representation robustness and reducing dependence on annotation (37).

Following intramodal encoding, information fusion across modalities is widely regarded as a central step for constructing unified representations (22, 38). In this review, we group mainstream fusion strategies into three broad families: early, intermediate, and late fusion. Early fusion combines raw or low-level features from different sources and is best suited to closely related modalities, but offers limited flexibility for modelling complex semantics. Late fusion maintains separate modality-specific models and aggregates their outputs at the decision layer via ensembling, stacking, or mixture-of-experts schemes; this modular design is straightforward to integrate into clinical workflows and relatively tolerant to missing modalities, but supports only shallow cross-modal interaction. Intermediate fusion operates at the representation level and encompasses a wide spectrum of architectures—including shared embedding spaces, co-attention blocks, and cross-attention Transformers—that enable richer non-linear interactions between heterogeneous modalities. Table 1 provides a structured comparison of these three fusion families, summarising their typical architectural patterns, strengths and limitations, and robustness to missing modalities.

**Table 1:** Comparison of multimodal fusion strategies in medical AI.

Fusion strategy	Typical architecture	Main strengths	Main limitations	Typical use cases/data conditions
Early fusion	Feature concatenation, shared encoder	Simple; preserves raw feature information	Sensitive to noise; poor with heterogeneous modalities	Multi-sequence MRI
Intermediate fusion	Shared latent space, co-attention, cross-attention Transformer	Interactive; captures fine-grained cross-modal dependencies	High training cost; requires well-paired data	Radiology/pathology + clinical text
Late fusion	Ensemble, stacking, mixture-of-experts	Modular; handles missing modalities well	Shallow interactions	Imaging + EHR; Radiology + pathology

In many recent systems, auxiliary mechanisms such as contrastive learning frameworks (39–44), modality alignment networks (45–49), modal adapters (50, 51) are layered on top of these three fusion families to enforce semantic consistency and allow large pretrained models, particularly Transformers, to incorporate new modalities with limited retraining.

Beyond these fusion strategies, some studies also explore cross-modal learning to associate imaging morphology with molecular or physiological signals, helping to reveal phenotype–genotype relationships, although this typically requires carefully paired data and raises additional annotation and ethical challenges (52–54).

In practice, multimodal clinical datasets often contain partially missing or asynchronous inputs, and many recent studies address this directly at the model level. From a fusion perspective, common strategies include temporal-alignment schemes that map sparse modalities (such as intermittent imaging) onto a unified timeline using nearest-neighbour or carry-forward assignment (55) with explicit masking; modular late- or mixture-of-experts architectures (56) whose branches can operate independently so that predictions remain available when some inputs are absent; and robustness-oriented mechanisms—most commonly attention-based reweighting (57) and modality dropout (58)—to reduce over-reliance on any single source and to down-weight noisy or unavailable modalities. Across the studies we reviewed, modular late- or hybrid-fusion designs, together with intermediate-fusion models equipped with such robustness mechanisms, appear particularly tolerant to missing modalities in practice, although no single strategy is universally optimal across tasks.

**Figure 2:**
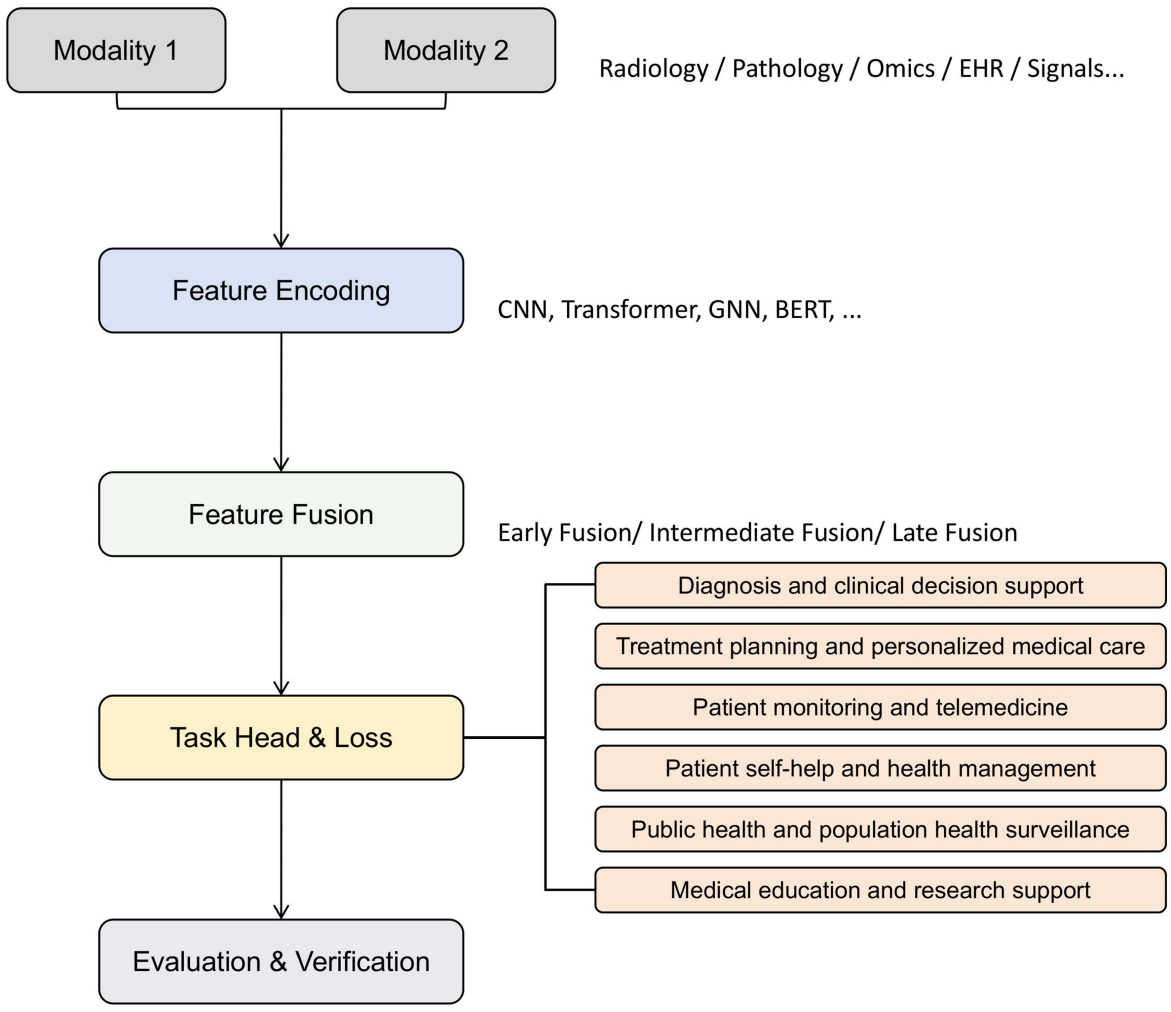
Task-oriented methodological framework for multimodal medical AI. Heterogeneous inputs are encoded and fused (early, intermediate, late) to support six core tasks—diagnosis, treatment planning, monitoring, self-management, public health, and education—evaluated by standard metrics and clinical validation.

To link these methodological components to clinical utility, we introduce a task-oriented methodological framework (Figure 2), illustrating the logical progression from data acquisition, encoding, and fusion to task-specific modeling and evaluation. By explicitly aligning technical processes with clinical objectives such as diagnosis, treatment planning, and health management, this framework provides both a structured lens for interpreting existing studies and a roadmap for identifying research gaps and future directions.

## Model architectures and clinical functionality

In multimodal medical AI systems, the model architecture plays a pivotal role not only in determining the pathways of information flow and the strategies for modality fusion, but also in shaping the system’s applicability and deployment flexibility within clinical settings. Architectural design is typically guided by the specific objectives of the clinical application and the availability and characteristics of the involved data modalities. As evidenced by the reviewed literature, most existing models share a common construction paradigm comprising three core components: feature extraction, inter-modal fusion, and task-specific output generation.

During feature extraction, data from different modalities are processed through independent encoding pathways to capture distinct structural and semantic features. Recent adoption of Transformer architectures has demonstrated superior global modeling capabilities compared to traditional convolutional models across various tasks (59). Meanwhile, convolutional neural networks (CNNs) remain the dominant approach for image modalities, given their spatial feature extraction capabilities. For structured clinical indicators and low-dimensional data, traditional machine learning methods continue to be used for initial feature extraction and selection, reducing model complexity and improving computational efficiency (60–62).

The task output module plays a pivotal role in translating model capabilities into specific clinical functions, influencing both prediction accuracy and the scope of healthcare processes it supports. Multimodal AI models are increasingly applied to diverse clinical tasks such as early disease screening, automated diagnosis, prognosis estimation, and treatment decision support. These tasks are operationalized through various output mechanisms—such as categorical classification, risk scoring, or structured text generation—addressing multilevel clinical needs from lesion assessment to global patient evaluation. With growing clinical complexity, multi-task architectures are emerging as a mainstream approach to enhance system adaptability across healthcare workflows (63–65).

Beyond structural performance, model interpretability (60–62) and human-computer interaction (HCI) capabilities (66, 67) are critical to architectural design. Recent advancements have enhanced AI transparency through attention visualization (68, 69), feature response mapping (70), and semantic pathway prompting (69, 71), while interactive interfaces allow clinicians to adjust and intervene in model behavior, enhancing informed decision-making (72–74). These strategies build clinician trust and promote physician-AI collaboration, ultimately improving real-world applicability. Overall, the architectural design of multimodal medical AI systems is evolving toward greater flexibility and alignment with clinical needs.

## Multimodal AI applications by clinical task categories

Classifying multimodal AI research by targeted clinical tasks provides a systematic framework for evaluating its application value and developmental trajectory in real-world medical practice. This task-oriented perspective enables a comprehensive understanding of current trends and clarifies how different clinical scenarios impose distinct requirements on model architecture, data fusion, and performance evaluation. The specific needs of each task—such as modality weighting, feature interaction, and clinical validation—collectively define the core scientific and technical challenges in multimodal medical AI.

In this section, existing multimodal AI research is categorized according to clinical task dimensions, encompassing major application domains such as diagnosis and decision support, treatment planning and personalized care, patient monitoring and telemedicine, self-management and health maintenance, public and population health, and medical education and research. Studies outside these categories are summarized as “Other medical applications.” For each task category, we review representative research paradigms, methodological advances, and practical implementation models to outline technological pathways and innovation directions across diverse clinical contexts.

This task-oriented framework highlights that seemingly distinct clinical problems often share common technical requirements. For example, ICU monitoring, surgical navigation, and blood glucose prediction—though differing in context—all involve challenges in real-time inference, temporal modeling, and uncertainty handling. Analyzing the field through this lens helps identify generalizable solutions that can be translated across domains, thereby accelerating progress toward an intelligent healthcare ecosystem.

## Diagnosis and clinical decision support

Existing research has focused on two core clinical application scenarios: diagnosis and clinical decision support (22). Regarding the disease spectrum, current studies are predominantly focused on two categories characterized by significant clinical challenges: oncological diseases and neurodegenerative disorders. These conditions have emerged as prominent areas for the application of multimodal AI technologies due to their intricate pathophysiology, substantial heterogeneity, and the clinical challenges associated with early-stage diagnosis.

At the technical implementation level, imaging modalities constitute the primary data source in most studies. However, disease-specific variations exist in the choice of imaging techniques: tumor diagnosis typically relies on functional-anatomical hybrid imaging, such as CT or PET/CT, whereas neurodegenerative disease assessment primarily utilizes MRI as the core modality. Notably, mainstream approaches tend to adopt a multimodal fusion framework that integrates imaging features with clinical variables and histopathological data—either in pairs or all three synergistically. This integrated modeling strategy has been shown to significantly enhance diagnostic accuracy and disease subtype differentiation, underscoring the technological strengths of multimodal AI in addressing complex clinical challenges.

**Figure 3:**
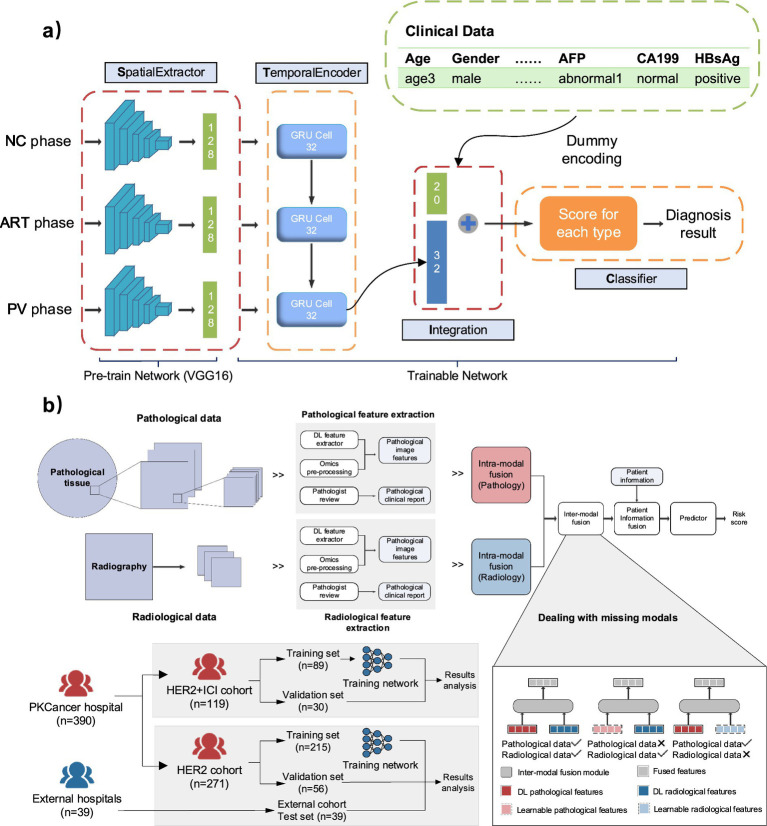
**(a)** Modular structure of the STIC deep learning framework for hepatic tumor classification. **(b)** Workflow of MuMo, integrating multimodal imaging and clinical data to predict anti-HER2 therapy response in gastric cancer with robustness to missing modalities. Panel **(a)** adapted from Gao et al. (75) and panel **(b)** from Chen et al. (76), both licensed under the Creative Commons Attribution 4.0 International License (CC BY 4.0: https://creativecommons.org/licenses/by/4.0/).

In recent years, multimodal AI has achieved substantial progress in the diagnosis of malignant tumors and clinical decision-making. Its applications now span major cancers, including liver, gastric, breast, and lung malignancies. Among these, liver cancer remains one of the most prevalent globally. Traditional diagnostic approaches, which rely on manual interpretation of radiological images combined with clinical evaluation, often fall short in early-stage or atypical cases, leading to misdiagnosis. To address these limitations, Gao et al. (75) developed STIC (Spatial Extractor-Temporal Encoder-Integration-Classifier), a deep-learning framework integrating multiphase contrast-enhanced CT images with clinical data. By jointly modeling spatial features, temporal dynamics, and clinical indicators, STIC effectively discriminates among three liver-cancer subtypes—hepatocellular carcinoma (HCC), intrahepatic cholangiocarcinoma (ICC), and hepatic metastasis—achieving 86.2% accuracy for HCC-ICC differentiation and 82.9% for three-class classification on external validation (Figure 3a). This study highlights how combining imaging-based spatial–temporal representations with clinical knowledge improves diagnostic generalization and supports precise liver-cancer stratification.

Gastric cancer is a lethal gastrointestinal malignancy characterized by high heterogeneity in therapeutic response, complicating clinical management. This challenge is particularly evident in HER2-positive patients, where optimizing targeted and immunotherapy combinations remains difficult. Conventional efficacy assessments based on imaging interpretation and molecular subtyping often fail to predict individualized treatment outcomes. Chen et al. (76) developed MuMo, a multimodal deep-learning framework that integrates contrast-enhanced CT, whole-slide histopathology, and structured clinical data through a two-stage intra- and inter-modal fusion mechanism. MuMo achieved AUCs of 0.821 for anti-HER2 monotherapy and 0.914 for combined therapy, substantially outperforming unimodal baselines (Figure 3b). Survival analysis further revealed prolonged progression-free and overall survival in the MuMo-predicted low-risk group. This study demonstrates the potential of multimodal AI to guide precision treatment selection and prognostic assessment in HER2-positive gastric cancer. This work compellingly demonstrates the promise of multimodal AI for precision treatment selection in HER2-positive gastric cancer, showing clear performance gains over unimodal approaches and highlighting the value of integrating imaging, pathology, and clinical data. Looking ahead, the broader clinical impact of such systems will depend on validation across larger and more diverse cohorts, as well as improved robustness to variations in imaging protocols and tissue preparation. Strengthening interpretability and aligning model outputs more closely with real-world treatment pathways will also be essential steps toward enabling routine clinical adoption. Representative multimodal systems for cancer diagnosis and clinical decision support are summarized in Table 2.

**Table 2:** Representative applications of multimodal AI in cancer diagnosis and clinical decision support.

Objectives	Methods	Fusion strategy	Modals	Results
Glioma	Deep neural networks (DNN), ensemble clustering algorithms	Intermediate fusion (feature-level integration), late fusion (ensemble)	MRI, WSI, WES, RNA-seq, Clinical Data	The proposed model MOFS demonstrated improved prognostic prediction, significant survival stratification (*p* < 0.001), and high performance in imaging-based subtype(MOFS1, MOFS2, MOFS3) prediction (AUCs = 0.889–0.968) (135).
Breast cancer	CNN, temporal encoding (3D Conv + position embedding + transformer encoder), logistic regression	Intermediate image fusion, late fusion with clinical data	X-ray, MRI, EHR	The proposed MRP model outperformed radiologists by up to 37% in AUROC for pre-treatment response prediction and demonstrated potential to reduce treatment toxicity by 35.8% and surgery rates by 16.7% (136).
Ovarian cancer	CNN (ResNet-18), Transformer, ViT, MLP	Intermediate fusion (modality-specific + transformer-based fusion)	B-mode and colour Doppler images, O-RADS scores, clinical factors	The proposed OvcaFinder model achieved AUCs of 0.978 (internal) and 0.947 (external), and reducing radiologists’ false positive rates by 13.4 and 8.3%, respectively (86).
Nasopharynx cancer	CNN, MLP, Transformer	Intermediate fusion, Late fusion	MRI, Clinical biomarkers, Blood tests, Lifestyle factors	The proposed model achieved competitive performance across three centers, with a peak C-index of 0.789, and maintained robust prognostic accuracy under up to 100% modality missing rates (87).
Esophageal cancer	CNN, SVM	Late fusion	Contrast-enhanced CT radiomics, WSI pathomics, clinical variables	The multimodal model achieved an AUC of 0.89 on the test set, outperforming all single-modal models and offering interpretability via SHAP for personalized preoperative decision-making (130).
Colorectal cancer	DNN, GMM, LASSO, Random Forest classifier	Late fusion	PET/CT imaging, clinical data, IHC biomarkers	The proposed model DERBY+ achieved AUCs of 0.95 (internal) and 0.83 (external) in predicting bevacizumab treatment response in colorectal-cancer liver metastases (88).
Skin cancer	Vision Transformer (ViT), MLP, Cross-modal contrastive learning and joint pretraining	Hybrid fusion (contrastive alignment + joint embedding + downstream tuning)	Total-body photographs (TBP), Clinical images, Dermoscopic images	The proposed model PanDerm significantly improved clinicians’ diagnostic accuracy for melanoma from 0.69 to 0.83, with the model alone achieving an accuracy of 0.81, comparable to clinician-AI collaboration (82).

Recent advances in multimodal AI have markedly improved the early diagnosis, subtype stratification, and prognosis prediction of neurodegenerative disorders such as Alzheimer’s disease (AD) and Parkinson’s disease (PD), consistently outperforming unimodal approaches and offering more reliable decision-support tools in clinical practice.

Alzheimer’s disease is a progressive condition marked by cognitive decline and substantial heterogeneity. Accurate identification requires integrating neuroimaging, molecular biomarkers, and neuropsychological data, as traditional unimodal methods—such as structural MRI or brief cognitive tests—show limited sensitivity in prodromal stages. To address this limitation, Lei et al. (77) proposed FIL-DMGNN, which combines Feature-Induced Learning with a Dual Multilevel Graph Neural Network to fuse genomic, MRI, proteomic, and clinical features. Tested on the ADNI dataset, FIL-DMGNN surpassed benchmark models across multiple metrics, effectively distinguishing clinical stages of AD and demonstrating the value of cross-scale integration for precision diagnosis. However, despite its strong performance on the well-curated ADNI dataset, such approaches rely heavily on complete multi-omics and imaging profiles. In typical clinical settings, data sparsity, missing modalities, and the cost of molecular testing remain major barriers, so it is still uncertain whether these gains can be reproduced in real-world populations.

Parkinson’s disease also exhibits strong inter-individual heterogeneity in its clinical trajectory. Su et al. (78) developed a longitudinal multimodal framework integrating serial clinical data, MRI, RNA-seq, and genotype information for 90 PD-associated SNPs, augmented with protein–protein interaction (PPI) networks. Applied to the PPMI cohort, the model identified three progression subtypes (PD-I, PD-M, PD-R) and achieved a mean AUC of 0.87 for distinguishing the rapidly progressing PD-R subtype. Network analyses revealed subtype-specific molecular modules and suggested repurposable therapies, with real-world validation supporting the potential of metformin and similar compounds to mitigate cognitive and motor decline in PD-R patients.

The emergence of large language models (LLMs) is reshaping diagnostic and decision-support paradigms. Within pretraining-finetuning frameworks, LLMs exhibit strong cross-modal integration, processing heterogeneous data such as EHRs, radiology reports, and genomic profiles. Their attention-based architectures enable contextual reasoning comparable to expert-level performance in differential diagnosis and treatment planning. Sandmann et al. (79) evaluated the open-source model DeepSeek-R1 on diverse clinical tasks—USMLE-style examinations, NEJM case analyses, RECIST 1.1 tumor-response classification, and radiology-report summarization—achieving 92% accuracy on USMLE questions, F1 scores of 0.57–0.74 on narrative-case tasks, and an AUC of 0.74 for tumor-response prediction. Expert assessments rated its diagnostic reasoning (3.6/5) and summary quality (4.5/5) highly, underscoring its potential in clinical reasoning and multimodal analysis.

Beyond text-only reasoning, LLMs are increasingly applied to multimodal medical tasks that integrate images and clinical context. Oh et al. (80) introduced LLMSeg, which fuses a LLaMA-2-7B language backbone with a 3-D Residual U-Net image encoder for radiotherapy target delineation. By aligning visual and textual features within a shared semantic space, LLMSeg outperformed conventional image-only models, yielding higher Dice scores and better inter-expert concordance, particularly for complex breast-cancer contouring tasks. Nevertheless, the model’s reliance on detailed textual prompts and well-structured imaging data raises questions about its robustness in routine clinical workflows, where contouring instructions may be incomplete and imaging quality highly variable. Moreover, its performance has so far been validated only in controlled research cohorts, leaving its real-world generalizability and safety for clinical deployment uncertain.

Despite these advances, clinical deployment of AI systems faces persistent challenges in ethical governance, transparency, and clinician-AI collaboration. Dykstra et al. (81) proposed PULSE, an end-to-end framework covering patient consent, multimodal integration, and unified data governance. Validated on over 30,000 patient records, PULSE outlines a practical route toward fair, safe, and responsible AI implementation in healthcare. More broadly, recent multimodal systems have begun to address these concerns in practice by reporting stratified performance across demographic subgroups (82, 83) to assess potential bias and by employing federated learning or secure data enclaves (84) to limit raw data movement across institutions.

As multimodal AI systems move from research prototypes to bedside use, their value depends not only on gains in diagnostic accuracy but also on achieving interpretability, trustworthiness, and practical deployability through well-designed clinician-AI collaboration. Guided by this principle, several studies have adopted human-computer co-design frameworks to create interactive tools for real clinical settings. For instance, Zhang et al. (85) introduced SepsisLab for early sepsis detection. The system visualises predictive uncertainty and proactively recommends additional laboratory investigations, allowing physicians to recognise high-risk cases sooner and more accurately. In retrospective, offline evaluation on the MIMIC-III dataset that simulated a missing-data scenario, SepsisLab raised the model’s area under the ROC curve (AUC) by roughly ten percentage points relative to a missing-data baseline, while requiring only 9.6% extra key laboratory tests—performance nearly equivalent to that achieved with complete observations. These findings highlight the effectiveness of an active-sensing strategy in enhancing model performance without incurring substantial additional testing burden.

Taken together, current multimodal systems have shown clear benefits in diagnostic accuracy, subtype differentiation, and treatment-relevant risk stratification, particularly when modalities provide truly complementary information. Across studies, several recurring limitations also emerge. When models are evaluated across centers or populations, performance often degrades (86–88), reflected in lower AUC, reduced sensitivity for minority subgroups, and calibration drift due to domain shifts in imaging protocols, EHR coding practices, or patient mix. In addition, jointly using highly sensitive data—imaging, EHRs, and omics—raises privacy and governance challenges that require clearer consent processes and task-specific alignment with clinical workflows (89).

Methodologically, intermediate fusion enables richer cross-modal interaction but is more sensitive to missing or low-quality inputs; in contrast, modular late-fusion designs generalize more robustly across clinical settings because modality branches can function independently. A small number of studies incorporate uncertainty estimation or clinician-in-the-loop mechanisms, showing improved reliability in ambiguous or low-confidence scenarios.

Overall, while multimodal AI has demonstrated strong potential for enhancing diagnostic decision-making, its benefits are neither universal nor uniformly stable. Future progress will depend on robust cross-modal alignment, mechanisms for handling missing or heterogeneous data, clinically meaningful uncertainty communication, and tighter integration with real-world diagnostic workflows.

## Treatment planning and personalized medical care

Treatment planning and personalized medicine represent a challenging yet transformative frontier in clinical AI. Their success depends on integrating heterogeneous time-series data—from longitudinal EHRs and serial imaging to dynamic laboratory and multi-omics profiles—into unified representations that predict therapeutic response, complication risk, and prognostic trajectories. These goals demand models with strong generalizability, interpretability, and adaptive updating, driving advances in efficient fusion architectures and clinician-centered interaction frameworks.

In non-small-cell lung cancer (NSCLC), response to EGFR-targeted therapy such as osimertinib varies widely across patients owing to intrinsic and acquired drug resistance. Hua et al. (90) developed a multimodal model integrating whole-slide histopathology, somatic-mutation profiles, and clinical variables to predict resistance, achieving a mean concordance index (C-index = 0.82) and surpassing unimodal baselines. Explainability analyses identified RB1 mutations, nuclear-morphological abnormalities, and inflammatory microenvironmental features as key prognostic markers. Complementarily, Keyl et al. (91) constructed a deep-learning framework combining EHRs, CT-derived body-composition features, and tumor-mutation profiles. Across a pan-cancer cohort, the model robustly predicted survival endpoints—overall survival and time-to-next-treatment—while disentangling modality-specific contributions through an interpretability module. Together, these studies illustrate how multimodal integration enhances both predictive accuracy and biological plausibility in complex prognosis modeling.

**Figure 4:**
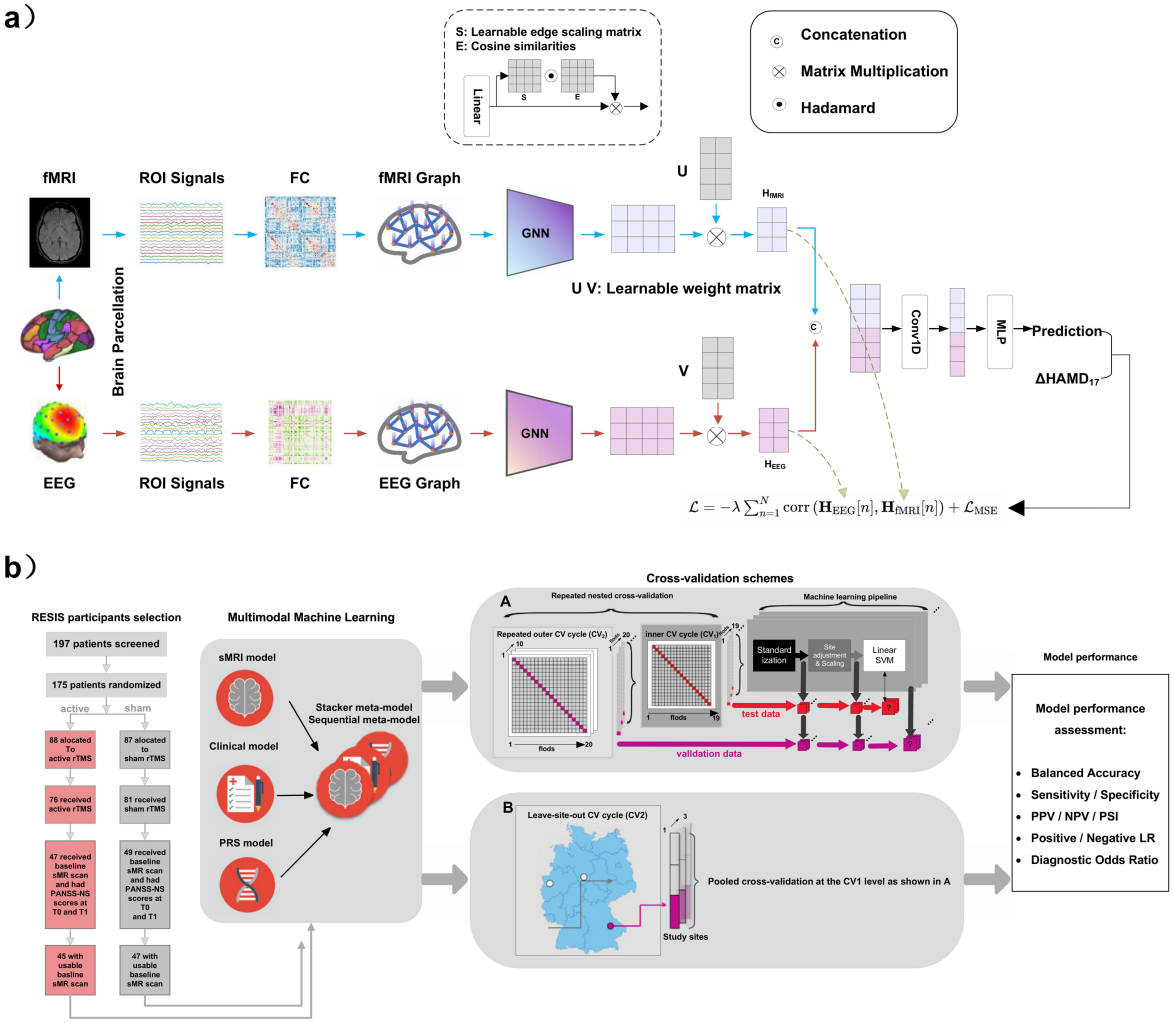
**(a)** Flowchart of the proposed multimodal prediction framework. Functional connectivity features from fMRI and EEG are encoded by parallel GNNs, projected onto latent variables, and fused for HAMD17 score change prediction using a 1D convolution and MLP. **(b)** Flowchart of the proposed multimodal machine learning framework for predicting rTMS treatment response in schizophrenia, integrating sMRI, clinical variables, and polygenic risk scores (PRS) through a sequential fusion approach, with model performance assessed via nested cross-validation and independent site validation. Panel **(a)** adapted from Jiao et al. (92) and panel **(b)** from Dong et al. (95), both licensed under the Creative Commons Attribution 4.0 International License (CC BY 4.0); changes were made (CC BY 4.0: https://creativecommons.org/licenses/by/4.0/).

In psychiatry, electroencephalography (EEG) has become a pivotal modality for individualized therapy prediction. Jiao et al. (92) introduced a graph-neural-network framework (Figure 4a) that fuses resting-state fMRI connectivity with EEG signals to jointly model spatial–temporal features, achieving R^2^ = 0.24 for sertraline and R^2^ = 0.20 for placebo—both outperforming unimodal baselines. Saliency analyses highlighted the inferior temporal gyrus, occipital visual cortex, and posterior cingulate cortex as key predictive loci. In non-invasive neuromodulation, response to repetitive transcranial magnetic stimulation (rTMS) remains heterogeneous due to genetic and neuroimaging variability (93, 94). Dong et al. (95) developed a sequential multimodal framework (Figure 4b) that incrementally integrates clinical data, polygenic-risk scores, and structural MRI features from multicenter cohorts. The model achieved 93.5% balanced accuracy in predicting rTMS response in schizophrenia, establishing a scalable paradigm for personalized neuropsychiatric interventions. Although encouraging, this approach still depends on multimodal inputs that may not be consistently available across clinical settings, indicating the need for further validation in broader and more diverse cohorts.

Recent advances in deep reinforcement learning and LLMs are catalyzing increasingly sophisticated applications of artificial intelligence to complex clinical treatment planning. In the intensive-care domain, Ma et al. (96) proposed the DAQN model, which fuses vital signs, laboratory results, and SOFA scores within an attention-enhanced Q-learning framework. DAQN achieved a weighted doubly-robust score of 0.35 on MIMIC-III—surpassing DRQN (0.24), DQN (0.17), and historical clinician policies (0.07)—suggesting meaningful potential for improving decision quality. Additionally, Zhao et al. (97) introduced FUAS-Agents, an MLLM-based system that integrates MRI with clinical variables to autonomously generate focused-ultrasound ablation plans, receiving expert ratings above 82% for completeness and accuracy in a prospective study. While these systems illustrate the promise of multimodal and agent-based approaches for complex treatment workflows, their reliance on offline evaluation, controlled datasets, and limited prospective testing indicates that substantial validation and safety assurance are still required before routine clinical adoption.

Existing studies in treatment planning frequently integrate pathology slides, imaging, genomics, and electronic health records (EHRs) to predict therapeutic response and prognosis, often leveraging sequential or attention-based fusion strategies.

Nevertheless, progress in this area is constrained by limited and heterogeneous response labels, the absence of dynamic model updating mechanisms, and persistent barriers to inter-institutional data sharing, which collectively hinder clinical translation and scalability.

Future research will likely benefit from the development of transferable foundation models with continual learning capabilities, the application of reinforcement learning frameworks for personalized therapy optimization, and the design of multimodal large language model (LLM)-driven decision platforms to support adaptive treatment planning.

## Patient monitoring and telemedicine

As healthcare delivery shifts from hospital-centric to continuous out-of-hospital care, patient monitoring and telemedicine have become primary arenas for multimodal AI. These applications demand real-time acquisition and dynamic inference across heterogeneous data streams—including medical images, physiological time-series signals, EHRs, and patient-reported symptoms. Addressing this challenge requires computational frameworks capable of temporal-dependency reasoning, clinical interpretability, and real-time responsiveness, thereby supporting critical remote-care decisions such as postoperative rehabilitation management, longitudinal surveillance of chronic disease, and critical illness warning. In recent years, the broad deployment of wearable sensors, home-health platforms, and smart devices has catalyzed the adoption of multimodal learning techniques, accelerating the maturation and large-scale implementation of telemedicine infrastructures.

**Figure 5:**
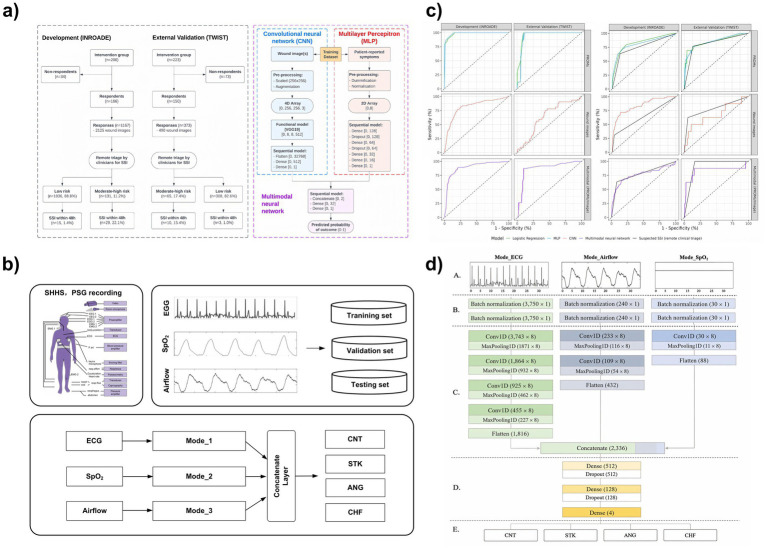
**(a)** End-to-end pipeline for multimodal data collection and labeling for SSI prediction (remote triage for suspected SSI; in-person assessment for confirmed SSI). **(b)** Diagnostic performance of multimodal assessment (wound images + patient-reported symptoms), consistently outperforming unimodal baselines for both suspected and confirmed SSI. **(c)** Multimodal CVD screening framework using PSG signals (ECG, airflow, SpO_2_) from SHHS processed by SleepCVD-Net. **(d)** SleepCVD-Net architecture with parallel 1D-CNN feature extractors, fusion, and four-class CVD classification. Panels **(a,b)** adapted from McLean et al. (100) and panels **(c,d)** from Kim et al. (103), both licensed under CC BY 4.0; changes were made (CC BY 4.0: https://creativecommons.org/licenses/by/4.0/).

Postoperative wound infection (SSI) is one of the most common complications of surgical procedures, which not only significantly increases the readmission rate and healthcare costs, but also affects the clinical prognosis (98). Conventional surveillance—typically outpatient or telephone follow-up—demands substantial clinical resources, suffers from incomplete data capture, and is prone to reporting bias, which collectively hamper early SSI detection (99). To mitigate these limitations, McLean et al. (100) developed a multimodal deep-learning model that fuses wound photographs with patient-reported outcome measures (PROMs) to enable remote SSI prediction (Figures 5a,b). In the prospective, external TWIST cohort, the model achieved an AUC of 0.834, matching or slightly exceeding clinician triage based on photographs alone (AUC = 0.784) while delivering comparable diagnostic accuracy. Workflow simulation further indicated that clinician review time per 100 patients would fall from 51.5 h to 9.1 h with model integration, showcasing a substantial reduction in manual workload and providing a robust foundation for intelligent, postoperative remote-monitoring systems.

Accurate seizure surveillance is essential for disease management, yet traditional single-modality monitoring—especially approaches relying solely on non-EEG physiological signals—often fails to detect focal seizures that lack overt motor signs (101). To overcome these limitations, Nielsen et al. (102) introduced a tri-modal wearable platform integrating ear-EEG, ECG, and accelerometry. The system achieved 84% sensitivity for focal tonic seizures and 100% for non-motor seizures at a false-alarm rate of 5–13 events per day, illustrating how complementary signals can substantially enhance real-world epilepsy monitoring. A similar trend is observed in cardiovascular disease (CVD) screening. While conventional models typically rely on unimodal daytime ECG or imaging, such inputs often fail to capture nocturnal physiological abnormalities that are clinically informative. Kim et al. (103) proposed SleepCVD-Net, which fuses ECG, airflow, and SpO_2_ from polysomnography (Figures 5c,d) and achieved 97.55% overall accuracy, with subtype F1 scores exceeding 96%. These results highlight the practical value of multimodal nocturnal monitoring for early cardiovascular-risk identification. Together, these studies demonstrate the clear advantages of multimodal physiological fusion for neurological and cardiovascular monitoring, while also underscoring remaining challenges such as sensor variability, patient adherence, and maintaining robustness outside controlled evaluation settings.

Beyond the continuous monitoring of epilepsy and cardiovascular diseases, multimodal AI also shows substantial promise in critical care. By integrating heterogeneous inputs—such as high-frequency physiological waveforms, EEG, medical imaging, and laboratory data—it enables continuous surveillance and dynamic risk assessment of ICU patients, markedly improving early-warning capabilities for neurological, circulatory, and respiratory deterioration (104). For instance, Zhang et al. (105) introduced the MANGO system, which fuses EHR data with wrist-accelerometry, facial-expression features, and environmental signals; in a cohort of 310 ICU patients, it achieved AUROC scores of 0.82 for severity prediction and 0.76 for life-support needs, outperforming EHR-only baselines. Boss et al. (106) developed the ICU Cockpit, a real-time platform that integrates more than 200 physiological waveforms, bedside videos, and laboratory results, demonstrating strong predictive performance in neurocritical-care deployment. While these studies highlight the potential of multimodal fusion to capture complex pathological events in the ICU, practical adoption will require addressing challenges such as heterogeneous data quality, sensor noise, and workflow integration to ensure robust real-world performance.

Monitoring and telemedicine systems increasingly integrate wearable devices with physiological signals (e.g., ECG, EEG, SpO_2_) and clinical data to facilitate remote risk assessment and continuous patient management.

Despite these advances, widespread adoption remains constrained by noisy or missing data, suboptimal patient adherence, and the computational burden of real-time analysis with explainability requirements. These challenges limit both the reliability and scalability of current solutions.

Future progress will depend on the development of lightweight, real-time models, novel approaches for heterogeneous temporal signal fusion, and closed-loop intelligent monitoring systems capable of delivering adaptive, individualized remote care.

## Patient self-help and health management

In the context of an aging population and the increasing burden of chronic diseases, patient self-management (PSM) has emerged as a crucial intervention strategy to improve disease control outcomes, enhance patients’ quality of life, and alleviate pressure on the healthcare system (107). Traditional health monitoring methods, which rely on regular outpatient follow-ups and patient self-records, have evident limitations, making it challenging to achieve continuous, dynamic, and multidimensional assessments of health status. In contrast, innovative monitoring systems based on multimodal AI technologies are driving significant changes in patient health management models by integrating multi-source, heterogeneous data, such as wearable device biosensor data, mobile health terminal information, structured electronic health records (EHRs), and voice/images.

**Figure 6:**
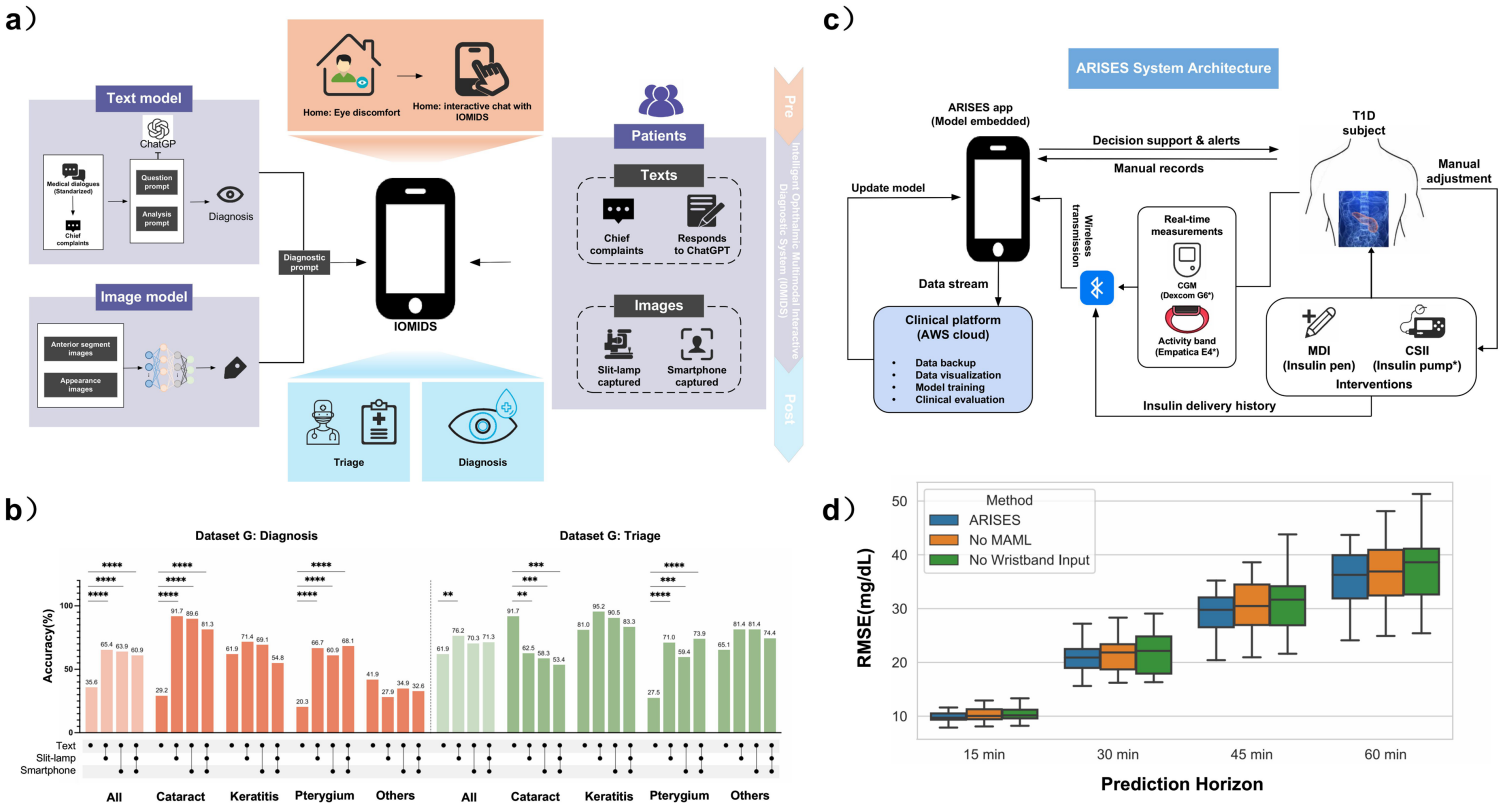
**(a)** IOMIDS architecture integrating ChatGPT-based text interaction with ocular image analysis for real-time diagnosis and subspecialty triage. **(b)** Overall and disease-specific diagnosis/triage accuracy across models in silent evaluation (Dataset G), showing gains from multimodal integration. **(c)** ARISES architecture combining CGM and wristband signals for personalized deep learning–based prediction in type 1 diabetes. **(d)** ARISES performance with MAML and wristband data across 12 subjects, showing reduced RMSE, with the largest improvement at the 60-min horizon. Panels **(a,b)** adapted from Ma et al. (18) and panels **(c,d)** from Zhu et al. (110), both licensed under the Creative Commons Attribution 4.0 International License (CC BY 4.0); changes were made (CC BY 4.0: https://creativecommons.org/licenses/by/4.0/).

With the rapid advancement of patient self-help health management technology and the widespread adoption of telemedicine services, the demand for at-home self-diagnosis and treatment among ophthalmology patients has significantly increased. This is primarily reflected in patients’ expectations for initial symptom identification and access to professional medical advice through convenient digital tools. However, current AI-based ophthalmic diagnostic systems have notable limitations: their model architectures are typically optimized for specific diseases, and their performance in cross-disease differential diagnosis remains insufficient, making it challenging to meet the growing demand for self-help consultation and accurate triage across diverse clinical scenarios (108). To address this challenge, Ma et al. (18) developed the IOMIDS system, which enables a multimodal self-service consultation feature that integrates text interaction with eye images captured via slit lamps or smartphones. This system combines a natural language processing engine based on the ChatGPT architecture with a deep learning image analysis model (Figure 6a). Experimental results demonstrate that the bimodal deep learning model, which combines text descriptions with eye images captured by smartphones, significantly outperforms the single-text input model. On a cross-center validation dataset, the diagnostic accuracy of the bimodal model reached 81.1%, a notable improvement over the 72.5% accuracy of the text-only model (Figure 6b). However, despite these gains, the system’s reliance on high-quality image capture and structured patient inputs may limit its consistency in real-world use, suggesting that further validation in broader home-care settings is still needed.

Type 1 diabetes mellitus (T1DM) accounts for 10% of global diabetes cases, with patients relying on insulin therapy and strict daily blood glucose management. While the use of continuous glucose monitoring (CGM) and insulin injection technologies has improved blood glucose control, significant challenges remain in self-management for T1DM patients due to individual differences in insulin response (pharmacokinetics/pharmacodynamics heterogeneity) and the impact of multiple endogenous and exogenous factors on blood glucose regulation (109). Building on this, Zhu et al. (110) proposed the ARISES system, which integrates continuous glucose monitoring (CGM) with multimodal physiological data collected from wearable wristbands, creating a mobile application platform embedded with deep learning models. This system enables real-time prediction of blood glucose trends and provides alerts for hypo- and hyperglycemic events (Figure 6c). An ablation study further demonstrates that incorporating wristband signals and the proposed lower-bound design consistently improves MCC for both hypoglycemia and hyperglycemia prediction across multiple time horizons (Figure 6d). Clinical validation studies show that, within a 30-min prediction window, the system achieved an average absolute error (MAE) of 24 in blood glucose predictions. In a 60-min prediction window, the system demonstrated an overall accuracy of 88.6% and a sensitivity of 70.3% for predicting hypoglycemia, significantly outperforming six baseline statistical or unimodal algorithms. This study confirms the clinical decision-support value of multimodal AI technologies in the self-management of chronic diseases such as diabetes.

In the clinical evaluation of multimodal AI wearable devices, patients’ subjective experiences and feedback play a crucial practical role. These health management systems achieve disease management through three core functional modules: continuous physiological monitoring, intelligent data analysis, and personalized interventions. The clinical effectiveness of such systems fundamentally depends on patients’ long-term adherence and engagement. Key psychological factors, such as patients’ acceptance of the technology, perceived usefulness, and ease of use, significantly influence the clinical adoption rate and willingness to continue using AI-assisted self-management tools. Therefore, Alzghaibi (111) conducted a questionnaire-based study involving 418 diabetic patients, utilizing a standardized questionnaire to assess patients’ perceptions and experiences with the use of an AI-integrated wearable device for diabetes self-management. The study results revealed that over 80% of participants acknowledged the positive effects of the technology on glucose monitoring, treatment adherence, and self-efficacy. However, three major barriers to usage were identified: concerns about data privacy (79.7%), the financial burden of the device (77.0%), and the complexity of using the technology (75.1%). The researchers emphasized the importance of considering user experience and technology acceptance when applying multimodal AI in patient self-management to enhance the effectiveness of practical applications.

However, their long-term effectiveness is limited by poor patient adherence, persistent concerns regarding privacy and data security, and restricted generalizability across different disease contexts, which collectively hinder widespread clinical adoption.

Future progress will likely depend on the development of multimodal interactive systems that integrate voice, images, and electronic health records (EHRs), the application of generative AI to enhance human-computer interaction, and the deployment of privacy-preserving personalized health assistants capable of sustaining engagement over time.

## Public health and population health surveillance

With the advancement of digital health technologies, public health and population health management are progressively transitioning toward a stage characterized by high-dimensional perception and intelligent responses (112). In contrast to individualized medical treatment, which primarily focuses on personal differences and precise interventions, public health emphasizes the integration of cross-population and multi-scenario data, along with the establishment of risk warning mechanisms. By synthesizing physiological signals collected from wearable devices, location data from mobile devices, climate and environmental exposure information, and other heterogeneous data sources such as electronic health records (EHRs), multimodal AI systems can facilitate large-scale, real-time monitoring of population health status and the modeling of disease trends. This population-centered intelligent surveillance framework not only enhances the responsiveness to public health emergencies but also provides crucial data to inform policy development and resource allocation (113).

Traditional screening methods for emerging infectious diseases encounter significant limitations in identifying asymptomatic or latent individuals. Therefore, there is an urgent need to establish a population-level early warning system that utilizes widely accessible health data, such as that from wearable devices, to enhance the sensitivity and efficiency of public health surveillance. Building on the DETECT (Digital Epidemic Exploration and Control Technology) project, Quer et al. (114) developed a multimodal COVID-19 intelligent monitoring framework that combines wearable sensor data with self-reported symptoms. A machine learning model was trained to predict COVID-19 nucleic acid test results by combining physiological signals, such as resting heart rate, step count, and sleep data, with self-reported symptom information from over 30,000 participants. The results showed that the fusion model outperformed each individual modality in distinguishing infection status, achieving an AUC of 0.80, which was significantly higher than models based solely on symptoms (AUC = 0.71) or sensor data (AUC = 0.72). Meanwhile, researchers have begun focusing on optimizing macro-level outbreak prediction by leveraging a broader range of data sources. Du et al. (115) proposed the PandemicLLM multimodal framework, which integrates heterogeneous data, including public policy texts, genomic surveillance, spatio-temporal outbreak profiles, and demographic-healthcare resources. These data are transformed into cue sequences that can be processed by a large language model, effectively reshaping real-time epidemic prediction into a ‘textual reasoning’ task. In a study covering 50 U.S. states over 19 consecutive months, PandemicLLM’s 1- and 3-week hospitalization forecasts outperformed the CDC Ensemble model in several error metrics. Furthermore, the inclusion of real-time genetic surveillance of mutant strains boosted the model’s prediction confidence by 20.1% and reduced the WMSE error by 28.2%. This suggests that the model can effectively utilize genomic surveillance data to enhance the accuracy and reliability of short-term epidemic predictions. However, the performance gains rely heavily on self-reported symptoms and participant compliance, suggesting that real-world scalability may be constrained by reporting bias and heterogeneous wearable usage patterns.

Since silent brain infarction (SBI) is frequently overlooked in routine clinical practice and is strongly linked to stroke, accurate and efficient detection of SBI is crucial for enhancing stroke risk prediction and optimizing public health intervention strategies. While magnetic resonance imaging (MRI) is considered the gold standard for detecting SBI, its application in clinical settings is often sporadic and delayed (116). Jiang et al. (83) introduced DeepRETStroke, a retinal-image–based framework for detecting silent brain infarction (SBI) and estimating stroke risk. Using a large self-supervised pre-training corpus (~900,000 images) and incorporating MRI labels and clinical features, the model achieved strong multicenter performance (AUC = 0.901 for first stroke; 0.769 for recurrent stroke) and enabled risk-stratified interventions that reduced recurrent stroke incidence by ~82%. These findings highlight the promise of low-cost retinal screening for population-level stroke prevention. However, the model’s dependence on large, standardized retinal datasets and high-quality label pipelines suggests that real-world scaling will require careful handling of device variability and heterogeneous screening workflows.

In the realm of regional disease prediction, Chen et al. (117) proposed a multimodal spatio-temporal prediction model based on LSTM networks, which integrates meteorological variables (e.g., precipitation, temperature, and humidity), historical incidence data, and geographic neighborhood relationships. The model also incorporates SHAP interpretable analysis to effectively capture the incidence trend, time lag, and spatial propagation characteristics of dengue fever across Brazil. The results demonstrated that the fusion model achieved the lowest MAE in 24 out of 27 Brazilian federal states, with the error in Amazonas and Pará reduced by approximately 40% compared to the baseline. When expanded to a 12-week forecast window, the model remained highly accurate in most states. SHAP interpretation revealed that relative humidity, minimum precipitation, and minimum temperature had the greatest influence, while the inclusion of case data from neighboring states further reduced the error by around 20%.

In addition, Wang et al. (118) introduced SatHealth, a multimodal framework that fuses satellite images, environmental variables, SDoH (Social Determinants of Health), and claims data for population-level disease prediction. The multimodal model achieved strong performance for regional diabetes incidence (*R*^2^ = 0.861) and improved 1-year individual risk prediction (AUROC = 0.815), demonstrating robust generalization across locations and time. A key consideration, however, is that such geographically rich models may face barriers to deployment due to variability in environmental measurements, social determinants, and region-specific data availability, which could limit transferability outside well-resourced settings.

Multimodal AI in public health increasingly integrates wearable sensors, environmental data, electronic health records (EHRs), policy documents, and genomic surveillance to enable epidemic forecasting and risk prediction.

Despite this potential, current progress is constrained by heterogeneous data standards, limited interpretability for policy translation, and the need to balance real-time responsiveness with predictive accuracy, which collectively restrict large-scale deployment.

Future advances will likely depend on harnessing large language models for cross-modal reasoning, establishing population-level risk prediction frameworks, and embedding AI-driven insights into public health decision-making systems to improve preparedness and responsiveness.

## Medical education and research support

In the context of the rapid development of medical artificial intelligence, multimodal AI technology is gradually demonstrating its unique value in the field of medical education and research. The traditional model of medical education, which relies primarily on textbook learning and clinical practice, faces several challenges, including limited access to quality case resources, an overloaded teaching workload, and significant individual differences in clinical experience. These challenges make it difficult to effectively address the needs of complex disease cognition and personalized skill training.

In medical education, multimodal generative AI offers a new approach to complex knowledge transfer and enhances the learning experience. Bland (119) developed the “Cinematic Clinical Narratives” framework, which integrates text, image, and speech generation models for teaching clinical pharmacology, creating a highly immersive teaching experience. The system, used for clinical pharmacology instruction, significantly boosted students’ contextual interest (with a mean rating of 4.58/5) and led to an average exam score of 88%. These results highlight the advantages of multimodal content in stimulating learning motivation and improving comprehension. Building on this, to further enhance interactivity and individual response perception during the learning process, Polo et al. (120) proposed and validated an emotion recognition system that integrates virtual reality (VR) with multimodal physiological signals (ECG, BVP, GSR, respiration) for medical simulation teaching and psychological research. The system achieved classification accuracies of 80% for potency and 85% for arousal across different emotional dimensions, and it identified GSR spikes as key emotional markers through interpretable AI mechanisms. Similarly, Xu et al. (121) introduced a well-designed multimodal psychological assessment framework that integrates facial video, speech-derived emotion cues, and interactive questionnaires. This work provides a clear demonstration of how dynamic multimodal signals can be leveraged to capture learner states more comprehensively than single-modality approaches, offering a valuable reference point for mental-health–related educational applications.

In the field of medical imaging education, a specialized area of technical training, Hossain et al. (122) proposed the Teach-Former framework, which leverages a knowledge distillation mechanism to fuse multimodal imaging data, such as CT, PET, and MRI, into a lightweight teaching model. The method achieved a Dice score of 0.766 on the automatic segmentation task of head and neck tumors (HECKTOR-21 dataset) and only showed a 1.5% reduction in Dice score compared to the integrated model consisting of three teacher networks in the prostate cancer detection task (PI-CAI dataset). At the same time, the number of model parameters and computation volume was reduced by 5.3 times and 10.8 times, respectively. The model significantly lowers computational requirements while maintaining accuracy, offering excellent portability and responsiveness. This makes it well-suited for medical imaging teaching and research platforms in resource-constrained environments, highlighting the potential of multimodal AI to enhance image interpretation capabilities and training efficiency in medical education.

Medical research is experiencing a paradigm shift, transitioning from being primarily experience-driven to becoming data-intensive and computationally empowered (123). The introduction of multimodal AI models not only enhances the accuracy of modeling complex systems but also drives the development of new research paradigms, including automated hypothesis generation, mechanism construction, and experimental optimization. Occhipinti et al. (124) proposed a mechanism-aware multimodal AI framework that integrates metabolic modeling, histology, and imaging data to make predictions that are not only accurate but also aligned with specific physiological mechanism pathways. This approach provides greater scientific insight and clinical adaptability compared to general model-uninterpretable methods such as SHAP or LIME. Based on this, the SpatialAgent proposed by Wang et al. (125) integrates LLM inference with 19 toolchains to simultaneously process four modalities: spatial transcriptome matrices, tissue section images, gene-pathway knowledge bases, and expert interactive text. This enables a one-stop solution for gene panel design, spatial locus annotation, and mechanism hypothesis generation. In the validation of Visium data, the auto-designed panel enhances cell type prediction accuracy by 6–19 percentage points compared to existing baseline algorithms. The MERFISH slice annotation achieves expert-level accuracy and significantly reduces analysis time. Moreover, it automatically generates mechanistic hypotheses for unknown data, showcasing the end-to-end discovery capabilities of multimodal AI agents in spatial biology research.

In education and research, multimodal AI increasingly integrates imaging, text, and speech to facilitate medical teaching, data annotation, and hypothesis generation.

However, progress in this domain is constrained by the absence of large-scale standardized educational datasets, limited interpretability, and difficulties in translating methodological advances into authentic clinical and academic environments, which together hinder sustainable impact.

Future progress will likely depend on the development of mechanism-aware multimodal frameworks, the use of multimodal-LLM agents for experimental design and hypothesis testing, and the creation of cross-disciplinary innovation platforms that can accelerate both medical training and research.

## Other medical applications

In addition to the core application scenarios mentioned above, multimodal AI technology has demonstrated significant potential in various emerging and interdisciplinary medical fields in recent years. The fusion of multimodal data not only broadens the scope of medical AI applications but also plays a crucial role in advancing precision medicine and systems medicine.

Among them, Demir et al. (126) proposed a multimodal AI framework for intraoperative stage recognition, integrating two modalities: speech (captured through three microphone channels—doctor, assistant, and environment) and medical imaging (intraoperative X-ray images and machine logs). The framework uses Gated Multimodal Units (GMUs) for feature fusion and employs a multistage Temporal Convolutional Network (MS-TCN) to model the temporal structure of the surgical procedure. In an experiment involving 28 port-catheter placements, the model achieved a frame-level accuracy of 92.65% and a macro-averaged F1 score of 92.30% for the surgical stage recognition task, marking an almost 10% improvement over previous methods. This study not only demonstrates the significant role of multimodal information in enhancing intraoperative recognition accuracy but also establishes a model for speech-driven intraoperative decision support systems.

In a preclinical drug development scenario, Partin et al. (127) proposed a multimodal neural network (MM-Net) framework that combines gene expression profiles with histological section images to predict tumor response to different drugs in a Patient-Derived Xenografts (PDX) model. The model integrates drug molecular descriptors, transcriptome expression, and digital pathology images, with its generalization capability enhanced by data augmentation strategies, including drug-alignment interchange and image enhancement. Validation results on the NCI PDMR dataset show that MM-Net outperforms unimodal baseline models in terms of the average Matthews correlation coefficient (MCC = 0.3102) and AUROC (0.7978), highlighting the feasibility and potential application of this strategy in preclinical drug screening. Additionally, multimodal AI demonstrates unique value in the highly challenging clinical task of rare disease diagnosis. Wu et al. (128) proposed and validated a multimodal model, GestaltMML, based on the Transformer architecture. This model fuses heterogeneous data, including facial images, demographic information, and clinical annotations from the Human Phenotype Ontology (HPO), to assist in the diagnosis of rare genetic diseases. Validated on the GestaltMatcher database, which includes 528 rare genetic diseases, as well as several typical syndromic datasets, the results showed that GestaltMML’s Top-10 diagnostic accuracy (83.6%) significantly surpassed that of the unimodal baseline model (72.4%), demonstrating its advantages in modeling phenotypic-genotypic associations.

In summary, multimodal AI technology is driving a paradigm shift in the medical field, expanding its application beyond traditional clinical diagnosis and treatment. It has demonstrated exceptional performance in the following key areas: (1) real-time surgical decision-making support, (2) innovative drug research and development, and (3) differential diagnosis of rare diseases. Through its robust multi-source information fusion and advanced cognitive reasoning capabilities, this technology offers crucial technical support for the development of a more comprehensive, accurate, and intelligent next-generation medical system.

To consolidate the evidence across these application domains, Table 3 provides a structured summary comparing representative multimodal models with their strongest unimodal baselines, including the few cases where multimodality does not confer performance gains.

**Table 3:** Comparative performance of multimodal models versus their unimodal baselines across representative clinical tasks.

Clinical task	Modalities (unimodal vs. multimodal)	Metric & performance (uni vs. multi)	Reference
Predicting gastric cancer HER2 response	CT-only vs. CT + WSI + clinical	AUC: 0.639 vs. 0.750	(76)
Predicting targeted therapy resistance in non-small cell lung cancer	Image - only vs. Image modality + Non-image modality	C-index: 0.75 vs. 0.82	(90)
Predicting surgical site infection	Wound image - only vs. PROMs + wound images	AUC: 0.671 vs. 0.834	(100)
Automated detection of tonic–clonic seizures	EEG - only vs. EEG + EMG + ACC	Sensitivity: 92.8% vs. 98.9%	(137)
Ophthalmic disease and subspecialty triage	Text - only vs. Text + images	Accuracy: 72.5% vs. 81.1%	(18)
COVID-19 detection	Wearable - only vs. Symptoms + wearable data	AUC: 0.72 vs. 0.80	(114)
Drug response prediction	GE - only vs. GE + WSI	MCC: 0.2958 vs. 0.3120	(127)

Across these domains, multimodal AI demonstrates strong capabilities in multi-source data fusion and advanced reasoning, providing critical support for next-generation intelligent medical systems.

At the same time, the survey reveals persistent methodological and translational bottlenecks, including challenges in data quality, generalizability, interpretability, and clinical adoption.

These collective observations motivate a forward-looking discussion on Future Directions, where technical advances, cross-disciplinary collaboration, and human-centered design will be essential to fully realize the transformative potential of multimodal AI in medicine.

## Discussion & future directions

Taken together, current multimodal medical AI systems have achieved meaningful performance gains in diagnosis, risk stratification, and monitoring across multiple tasks, but these improvements remain uneven across diseases and clinical settings, and persistent issues such as cross-center generalisation, bias, interpretability, and privacy continue to limit large-scale deployment. At the same time, it is important to note that these advantages are not universal: at least one published study has reported that adding an additional modality led to worse performance than the best unimodal model in a true multimodal clinical setting (129), suggesting that when an additional modality contributes little signal or substantial noise, the benefits of multimodality may be attenuated or even completely offset.

In this context, the task-oriented perspective introduced in this review offers a structured way to contextualise how multimodal methods function across different clinical scenarios. Rather than treating performance differences as purely model-driven, this perspective helps situate the potential contribution and limitations of each modality within the specific requirements of a given task. It provides a more organised lens for identifying recurring design patterns and potential bottlenecks across applications, and underscores the importance of aligning multimodal system design with concrete clinical objectives instead of relying solely on aggregate performance gains.

Building on the task-oriented perspective discussed in this review, a major frontier lies in transitioning from correlation-based fusion toward mechanism-aware integration—an evolution in which multimodal models learn not only to predict outcomes but also to infer the causal and physiological processes that generate them. Looking ahead, multimodal medical AI is expected to advance along several interrelated dimensions toward clinical translation and large-scale deployment.

At the algorithmic level, future studies should move beyond conventional fusion strategies toward adaptive, context-aware frameworks that dynamically adjust modality contributions. This requires semantic alignment across heterogeneous inputs and robust uncertainty quantification, supported by foundation models trained on large-scale multimodal datasets to enable transfer learning and generalization across tasks and diseases. Promising robustness-oriented designs include late- or hybrid-fusion schemes that preserve modality-specific encoders, site-aware normalization and domain-adaptation modules, uncertainty-aware regularization, and architectures that can gracefully handle missing or low-quality modalities.

At the system-design level, interpretability must be prioritized. From a practical standpoint, different data modalities tend to be paired with different families of explanation techniques: for medical images, saliency-based visualizations (82, 86, 130) such as Grad-CAM and attention heatmaps are widely used to highlight suspicious regions; for structured EHR variables and physiological time series, feature-attribution approaches (85, 130) (e.g., SHAP values or integrated gradients) are commonly applied to quantify the contribution of individual variables or time windows; textual inputs such as clinical notes and reports are often interpreted via attention-based or rationale-based explanations (79, 80); and omics or graph-structured data typically rely on pathway-level or node-importance analyses (78, 124) to relate predictions to underlying biology. Existing studies suggest that multimodal fusion does not inherently reduce interpretability, but it can make attribution more complex because contributions need to be disentangled across modalities and fusion layers. Future work should pursue intrinsic interpretability by embedding reasoning pathways, causal relationships, and domain knowledge within model architectures. The integration of knowledge graphs and causal inference can transform black-box systems into transparent decision-support tools, while human-in-the-loop designs will be essential for fostering trust and ensuring safe clinical deployment.

At the ecosystem level, collaborative multicenter networks and evolving regulatory frameworks are expected to play an essential role in translating multimodal AI into routine care. Such infrastructures should adopt harmonized data standards, federated learning, and privacy-preserving mechanisms, along with standardized validation pipelines encompassing data quality control, cross-institutional benchmarking, and clinical outcome assessment.

In parallel, major regulatory frameworks—such as the FDA’s *De Novo* and Breakthrough pathways, CE marking under the EU MDR/IVDR, UK MHRA guidance, and the forthcoming EU AI Act—are increasingly shaping how multimodal AI is translated into practice. Across these pathways, several requirements have become especially relevant for multimodal systems: a clearly defined intended-use statement, evidence of external validation across centers, transparent reporting of model behavior, and mechanisms for post-market monitoring. These expectations influence design choices by favouring modular fusion architectures that allow modality-specific validation, promoting uncertainty-aware outputs, and encouraging privacy-preserving training pipelines. Several multimodal AI systems have already been approved under these pathways, illustrating how regulatory frameworks shape real-world translation. For example, Canvas Dx (FDA *De Novo*) (131) integrates caregiver-recorded videos with standardized behavioral questionnaires for autism diagnosis; KidneyIntelX (FDA Breakthrough) (132) combines EHR variables with plasma biomarkers to stratify kidney-disease risk; Ibex Galen Prostate (CE-IVDR) (133) fuses whole-slide pathology images with clinical metadata to support cancer detection; and HeartFlow FFR-CT (FDA + NICE endorsement) (134) combines coronary CT imaging with computational modeling to guide treatment decisions. These deployed systems provide concrete evidence that multimodal AI can meet regulatory requirements and deliver measurable clinical utility—including reductions in diagnostic uncertainty, improved risk stratification, and more consistent treatment planning.

A small but growing number of multimodal systems are beginning to obtain regulatory clearance and enter pilot clinical use, yet future work must move beyond retrospective benchmarking toward prospective trials and real-world implementation studies that demonstrate tangible clinical benefit, workflow integration, and safety in everyday practice.

**Figure 7:**
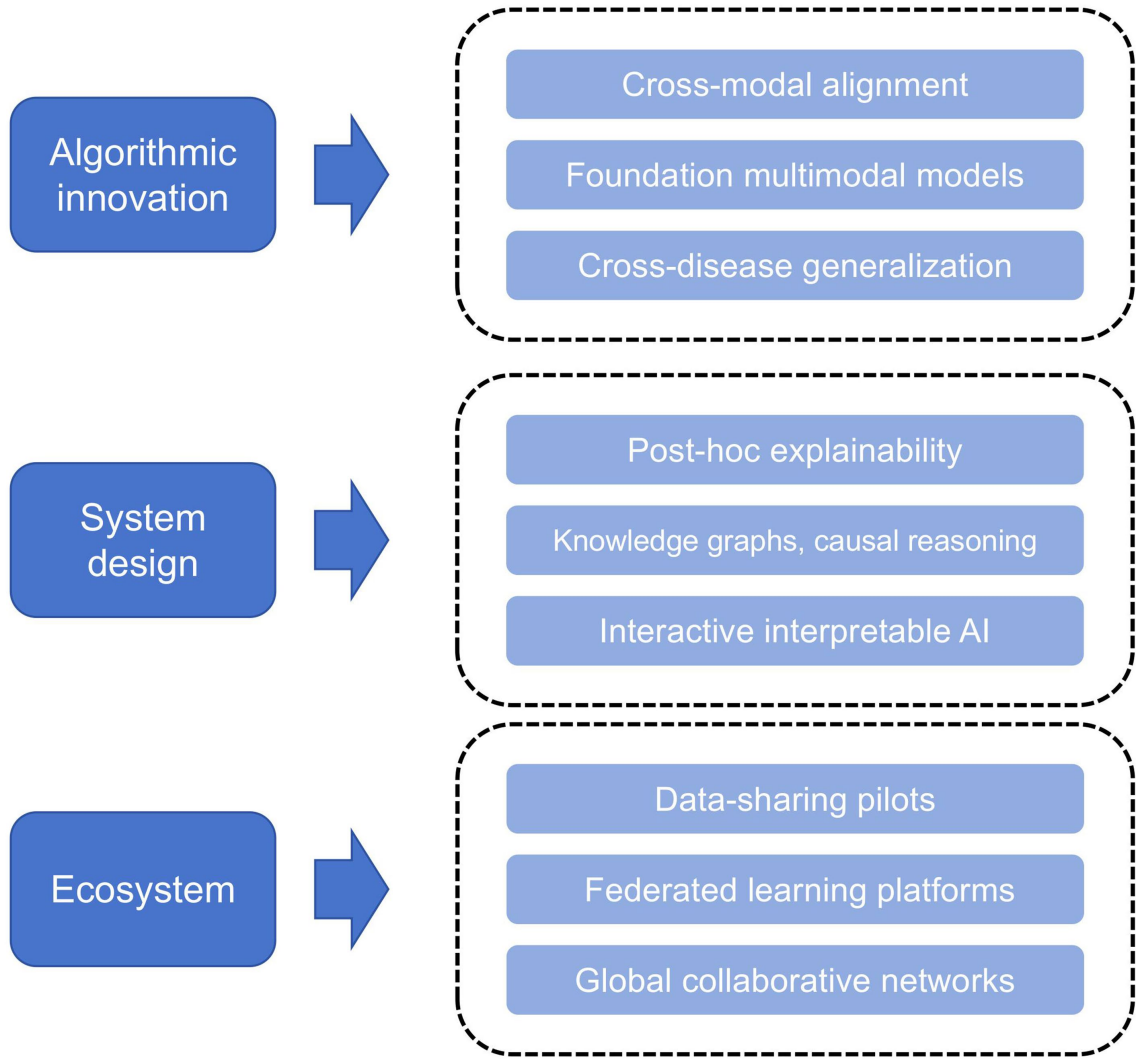
Future research roadmap for multimodal medical AI, which outlines progressive directions in algorithmic innovation, system design, and ecosystem development, emphasizing the methodological and infrastructural pathways needed for clinical translation.

Collectively, these directions extend the task-oriented framework proposed in this review, outlining a roadmap that connects methodological innovation, system design, and ecosystem development to the future of clinical translation (Figure 7).

## Conclusion

In this review, we synthesise recent advances in multimodal medical AI through a task-oriented framework that integrates methodological, clinical, and interdisciplinary perspectives. Beyond organising existing work, this framework provides a concise lens for evaluating current limitations and identifying paths toward clinical translation. It also clarifies where multimodality is most likely to offer added value and how methodological choices can be better aligned with concrete clinical objectives.

Across seven representative domains—diagnosis and decision support, treatment planning, monitoring and telemedicine, chronic disease self-management, public health, medical education and research, and frontier innovations—multimodal AI has often provided advantages over unimodal approaches. Beyond improved predictive performance (e.g., higher AUC and C-index), multimodal strategies have enabled novel clinical utilities such as real-time surgical guidance, AI-accelerated drug discovery, and rare disease diagnosis.

Nevertheless, progress remains uneven. Data heterogeneity, limited interpretability, cross-center generalizability, patient adherence, and regulatory uncertainty continue to impede large-scale deployment. These persistent challenges underscore that multimodal AI should be viewed not merely as a technical advance but as part of a socio-technical ecosystem that requires collaboration among computer scientists, clinicians, ethicists, policymakers, and patients.

Ultimately, multimodal medical AI represents more than incremental progress and contributes to a paradigm shift in how medicine is practiced, studied, and delivered. By consolidating methods, applications, and interdisciplinary insights, this review contributes a task-oriented synthesis that offers structured guidance for both current practice and future innovation. With sustained research, rigorous validation, and cross-disciplinary cooperation, multimodal AI has the potential to evolve into an important, clinically integrated and ethically grounded component of precision medicine and digital healthcare.
